# Protocol for a feasibility and acceptability study for UK general population paediatric type 1 diabetes screening—the EarLy Surveillance for Autoimmune diabetes (ELSA) study

**DOI:** 10.1111/dme.15490

**Published:** 2024-12-02

**Authors:** Lauren M. Quinn, Renuka P. Dias, Sheila M. Greenfield, Alex G. Richter, Joanna Garstang, David Shukla, Animesh Acharjee, Georgios Gkoutos, Richard Oram, Sian Faustini, Olga Boiko, Ian Litchfield, Felicity Boardman, Fatima Zakia, Christine Burt, Clair Connop, Amanda Lepley, Christine Gardner, Colin Dayan, Tim Barrett, Parth Narendran

**Affiliations:** ^1^ Institute of Immunology and Immunotherapy, College of Medicine and Health University of Birmingham Birmingham UK; ^2^ Department of Applied Health Sciences, College of Medicine and Health University of Birmingham Birmingham UK; ^3^ Department of Paediatric Endocrinology Birmingham Women and Children's Hospital Birmingham UK; ^4^ Clinical Immunology Service University of Birmingham Birmingham UK; ^5^ Birmingham Community Healthcare NHS Trust Birmingham UK; ^6^ School of Nursing University of Birmingham Birmingham UK; ^7^ Clinical Research Network, Lead for Primary Care (CRN West Midlands), National Institute for Health and Care Research Royal Wolverhampton Hospital Wolverhampton UK; ^8^ Institute of Cancer and Genomic Medicine University of Birmingham Birmingham UK; ^9^ MRC Health Data Research UK (HDR UK) Birmingham UK; ^10^ Institute of Translational Medicine University Hospitals Birmingham NHS Birmingham UK; ^11^ Centre for Health Data Research University of Birmingham Birmingham UK; ^12^ University of Exeter Exeter UK; ^13^ Warwick Applied Health, Warwick Medical School Warwick UK; ^14^ Community Connexions, Birmingham Community Healthcare NHS Foundation Trust Birmingham UK; ^15^ INVOLVE co‐applicant Birmingham UK; ^16^ Division of Infection and Immunity, School of Medicine University of Cardiff Cardiff UK; ^17^ Department of Diabetes University Hospitals of Birmingham Birmingham UK

**Keywords:** paediatrics, qualitative methods, screening, type 1 diabetes

## Abstract

**Aim:**

The EarLy Surveillance for Autoimmune (ELSA) study aims to explore the feasibility and acceptability of UK paediatric general population screening for type 1 diabetes.

**Methods:**

We aim to screen 20,000 children aged 3–13 years for islet‐specific autoantibodies through dried blood spot sample collection at home, hospital or community settings. Children with two or more autoantibodies are offered metabolic staging via oral glucose challenge testing. Feasibility assessments will compare recruitment modalities and uptake according to demographic factors (age, gender, ethnicity, level of deprivation and family history of diabetes) to determine optimal approaches for general population screening. The study is powered to identify 60 children (0.3%) with type 1 diabetes (stage 1–3). Parents are invited to qualitative interviews following ELSA completion (child screened negative or positive, single autoantibody or multiple, stage 1–3) to share their screening experience, strengths of the programme and any areas for improvement (acceptability assessments). Parents who decline screening or withdraw from participation are invited to interview to explore any concerns. Finally, we will interview professional stakeholders delivering the ELSA study to explore barriers and facilitators to implementation.

**Conclusion:**

Early detection of type 1 diabetes allows insulin treatment to be started sooner, avoids diagnosis as an emergency, gives families time to prepare and the opportunity to benefit from future prevention trials and treatments. ELSA will provide essential feasibility and acceptability assessments for UK general population screening to inform a future national screening programme for paediatric type 1 diabetes.

## 1 INTRODUCTION

Type 1 diabetes (T1D) is a chronic autoimmune condition resulting from immune‐mediated destruction of pancreatic islet beta cells, and a lifelong dependence on exogenous insulin therapy.^1^ Islet‐specific autoantibodies predate clinical T1D by 10–15 years.^2^ These autoantibodies can be detected in peripheral blood within the first few years of life.^3^ The presence of two or more islet autoantibodies indicates an almost lifetime certainty of future insulin‐requiring T1D.^2^ Children identified with two or more autoantibodies can subsequently be staged with oral glucose tolerance testing (OGTT): normoglycaemia (stage 1), dysglycaemia (stage 2), or stage 3 T1D, either asymptomatic (stage 3a) or insulin‐requiring (stage 3b).^4^ International guidelines recognise these early stages of T1D^5^ and diagnostic codes (SNOMED, ICD‐10) are now available for these presymptomatic stages (1 and 2).

The last decade has witnessed the emergence of international general population paediatric screening programmes for early detection of T1D, including Autoimmunity Screening for Kids (ASK) in the US and Fr1da in Germany.^6^ These programmes show that screening results in a significant reduction in presentation with diabetic ketoacidosis (DKA) at the time of T1D diagnosis, reducing DKA rates from up to 60% in some healthcare systems to less than 5%.^6^, ^7^, ^8^ Screening also identifies individuals who can benefit from immunoprevention trials and treatments for prevention.^4^ Teplizumab is currently licensed for T1D immunoprevention in the US and the UK National Institute for Health and Care Excellence (NICE) review of teplizumab is ongoing.^9^

Given that a licensed preventative treatment to delay T1D is a priority for stakeholders and healthcare professionals (HCP),^10^, ^11^ there is some urgency to establishing screening programmes to identify individuals with presymptomatic T1D in the UK. However, there are a range of social and ethical concerns surrounding early identification of incurable paediatric conditions and living with risk. These include potential for increased parental stress and loss of the child's right to ‘carefree’ time during the latent period of the disease which, in the case of T1D, could extend for many years. Thus far, questionnaire studies show that although screen‐detected T1D provokes anxiety at results notification, this dissipates after 12 months.^7^ We and others have also reported that parents value screening for DKA prevention, time to prepare and monitoring to facilitate a smooth transition to insulin requirement.^10^, ^12^, ^13^ However the broader acceptability and psychosocial implications of screening the general population and the most effective routes to approach and undertake the screening test within different populations in the UK healthcare system are unknown.

The overarching aim of the EarLy Surveillance for Autoimmune (ELSA) study is to assess the feasibility and acceptability of UK general population screening for T1D. Findings from ELSA will also contribute to the European action for the Diagnosis of Early Non‐clinical Type 1 diabetes For disease Interception (EDENT1FI) collaboration.

The specific objectives of the ELSA study are to:

1. Determine the best approach to recruitment into a surveillance programme for presymptomatic T1D through examining the feasibility of a variety of approaches, to access and include participants from diverse ethnicities and levels of deprivation.
2. Understand the perceptions and acceptability of families in the UK to be involved in an early detection programme for presymptomatic T1D, to establish how they would want to be informed and participate, and how any barriers to recruitment and participation can be addressed.
3. Understand the views of HCPs, school staff and other professional stakeholders involved in the testing programme to help understand the feasibility and acceptability of any future national screening programme.

## 2 METHODS AND ANALYSIS

### Study Population

All children aged 3–13 years inclusive, living in the four UK nations (England, Scotland, Wales and Northern Ireland) and not already identified with presymptomatic or insulin‐requiring T1D are eligible to participate in this study.

The 3–13 year age range was chosen for a number of reasons. First, the natural history of T1D is currently best characterised in children between pre‐school age (<5 years) and puberty (<14 years).^14^ Screening pre‐school children is particularly beneficial given the higher rates of DKA observed at T1D onset.^4^ Second, mapping a screening test onto an established public health programme ensures equitable access and costs. In the UK, the pre‐school measles, mumps and rubella (MMR) vaccination and 4‐in‐1 immunisations (diphtheria, tetanus, pertussis and polio) are offered at 3–4 years, and the human papillomavirus (HPV) vaccination is offered at age 12–13 years,^15^ thus facilitating access for screening purposes across these ages.

For the qualitative interviews, parent(s) or guardian(s) of children who completed the study, parents who withdrew or declined participation, and stakeholders involved in delivery of ELSA are invited to participate. Stakeholders include HCPs, teachers, headteachers, practice staff and study administrators. We aim to recruit a cohort of parents representative of the latest English Census.^16^

National Research Ethics Committee approval was obtained from Health and Research for Wales (Integrated Research Application System: 309252). International Standard Randomised Controlled Trial Number (ISRCTN): 97974414.

### 2.1 Sample size

We have based islet autoantibody UK seroprevalence on that identified in the Fr1da study of general population screening in Bavaria (0.3%).^7^ Fr1da is the largest general population autoantibody screening study and therefore offers the best estimate of autoantibody seroprevalence for European cohorts. If the data allows, we will assess UK seroprevalence according to demographic factors (age and ethnicity).

To allow sufficient numbers of participants for the qualitative interviews, it was determined that 20,000 children would need to be screened to identify 60 children at stages 1–3. Of these, we aim to interview 20–30 parents to reach thematic saturation.^7^ In addition, we aim to interview up to 10 parents who decline screening or withdraw and 20 professional stakeholders and will sample until thematic saturation is reached.^17^

A predesigned sampling grid for parents will enable acceptability assessments according to (1) demographics (age, sex, ethnicity, deprivation level and family history of diabetes), (2) recruitment modality (home testing, community or hospital) and (3) screening outcome (negative, false positive, single autoantibody, multiple autoantibodies, stage 1–3, withdrawn or declined). For stakeholders, we will purposively sample according to stakeholder role and workplace setting.

### 2.2 Informed consent and medical questionnaire

Information about the ELSA study is provided through leaflets and a study website (https://www.elsadiabetes.nhs.uk/). Information is provided in an age‐appropriate manner in English for children aged 3–6, 7–9 and 10–13 years. The parents' leaflet is translated into the ten most spoken UK languages.^16^ Parents are encouraged to make study‐related enquiries by either email, website or telephone. There is no financial incentive for participation.

The parent confirms eligibility, provides consent and selects their preferred testing location via REDCap (https://www.project‐redcap.org/), a secure web application for research databases (Supplementary File 1—Consent form). Children's assent (agreement to participate) is encouraged and governed by the parent. Medical information regarding family history of diabetes, and medical history of thyroid or coeliac disease are recorded. If symptoms of T1D are present or emerge during the study, parents are advised to contact appropriate medical services.

### 2.3 Recruitment

ELSA opened for recruitment in November 2022 in England and Wales, January 2023 in Scotland, and September 2023 in Northern Ireland. Diverse recruitment strategies across NHS clinical, community and home settings are being tested, in collaboration with the National Institute for Health Research's (NIHR) Clinical Research Networks (CRN) in England and Health Research for Wales and Scotland.

#### 2.3.1 Home testing

Home testing is available to families living in mainland Great Britain and Northern Ireland. Information about the ELSA study is disseminated via leading diabetes charities (Diabetes UK and Breakthrough T1D, formerly the Juvenile Diabetes Research Foundation), diabetes advocates, a health research recruitment service (https://www.nativve.com/) and social media.

Once informed consent is obtained via the online REDCap database, a home‐testing kit for dried blood spot (DBS) sample collection is dispatched along with an instructional leaflet (https://www.youtube.com/watch?v=uHHUJfHcJuY). The parent receives a text message confirming that a kit has been posted to them and the parent returns the completed kit via a stamped addressed envelope.

#### 2.3.2 Testing in NHS clinical settings

Invitations and up to two reminders are sent from general practices or NHS Trusts by text message, email, letter or telephone to parents with eligible children and study posters are displayed in clinical areas. Parents can consent online prior to attending the screening clinic or on arrival. The screening test is performed in clinical settings, including general practice, outpatient clinics or hospitals. In general practice, testing is offered alongside routine childhood immunisations. Outpatient clinics facilitate access to first‐degree relatives and testing feasibility and acceptability here is warranted as a potential first iteration of a UK national screening programme.

#### 2.3.3 Testing in community settings

UK schools are targeted given their experience in delivering childhood immunisations. NHS trusts and CRNs identify schools by first approaching the headteacher and approval may be sought from the school governing committee. Clinics are conducted on school premises, led by research delivery teams and supported by school staff.

Recruiting sites also target community organisations working with historically underserved communities, including refugees, ethnic minorities and in deprived regions. In collaboration with community leaders, an optimal clinic time and location are identified, and study information is shared beforehand. To overcome language barriers and low health literacy, translators are present to support informed consent.

#### 2.3.4 Qualitative recruitment and consent

On completion of the ELSA study, parents are invited to participate in a qualitative interview. There is an additional online consent form and demographics questionnaire. Parents who declined screening study participation are invited to interview via advertisements on social media and the study website, and stakeholders are invited directly from recruiting organisations.

### 2.4 Screening process

#### 2.4.1 Autoantibody screening test

**FIGURE 1:**
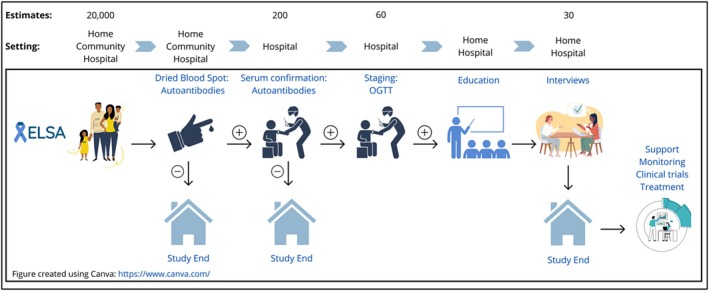
Summary of the ELSA Study. The child has a DBS performed either at home, in the community or at hospital. If this is negative, the child does not require further follow‐up. If the DBS is positive, a confirmatory venous collection is arranged. If this is positive for two or more autoantibodies, the child attends for an OGTT for type 1 diabetes staging. Parents of autoantibody positive (single, double or more) children are invited to an education session to inform about the signs and symptoms of type 1 diabetes and make them aware of research studies their child may be eligible for, including monitoring programmes and prevention studies. Parents are invited to participate in a qualitative interview following the education session. Figure created using Canva: https://www.canva.com/.

Figure 1 provides an overview of the study. DBS samples are collected from the child by the parent or HCP using a 2.0 mm Becton, Dickinson and company (BD) contact‐activated lancet (50 μL). DBS sampling has significant advantages over standard venepuncture, including small collection volume, less pain and easy sample collection with minimal training.^18^ Samples are returned within 24 h of collection to the UK Accreditation Service (UKAS) approved Clinical Immunology Service (CIS) laboratory (University of Birmingham). DBS samples are aliquoted within three days and stored at 4°C prior to analysis.

The DBS eluant is combination tested for anti‐glutamic acid decarboxylase (GAD), islet antigen 2 (anti‐IA‐2A) and zinc transporter 8 (ZnT8) autoantibodies using the 3‐screen multiplex RSR ELISA (3‐screen) (RSR Ltd., Cardiff, UK).^19^ The RSR LTD 3‐screen indicates a positive (≥20 IU) or negative result (<20 IU). We have validated the 3‐screen on DBS with a clinical sensitivity and specificity of 89% and 100% respectively (*manuscript in preparation*). These met or surpassed the manufacturers' guidelines for serum (sensitivity 86%, specificity 97%).^20^ The positive predictive value on DBS was 100% and negative predictive value was 71% (*manuscript in preparation*). If the 3‐screen is negative (< 20 international units (IU)), parents receive a text message with the negative result and a letter is sent to the parent and general practitioner (GP).

#### 2.4.2 Autoantibody confirmation test

**FIGURE 2:**
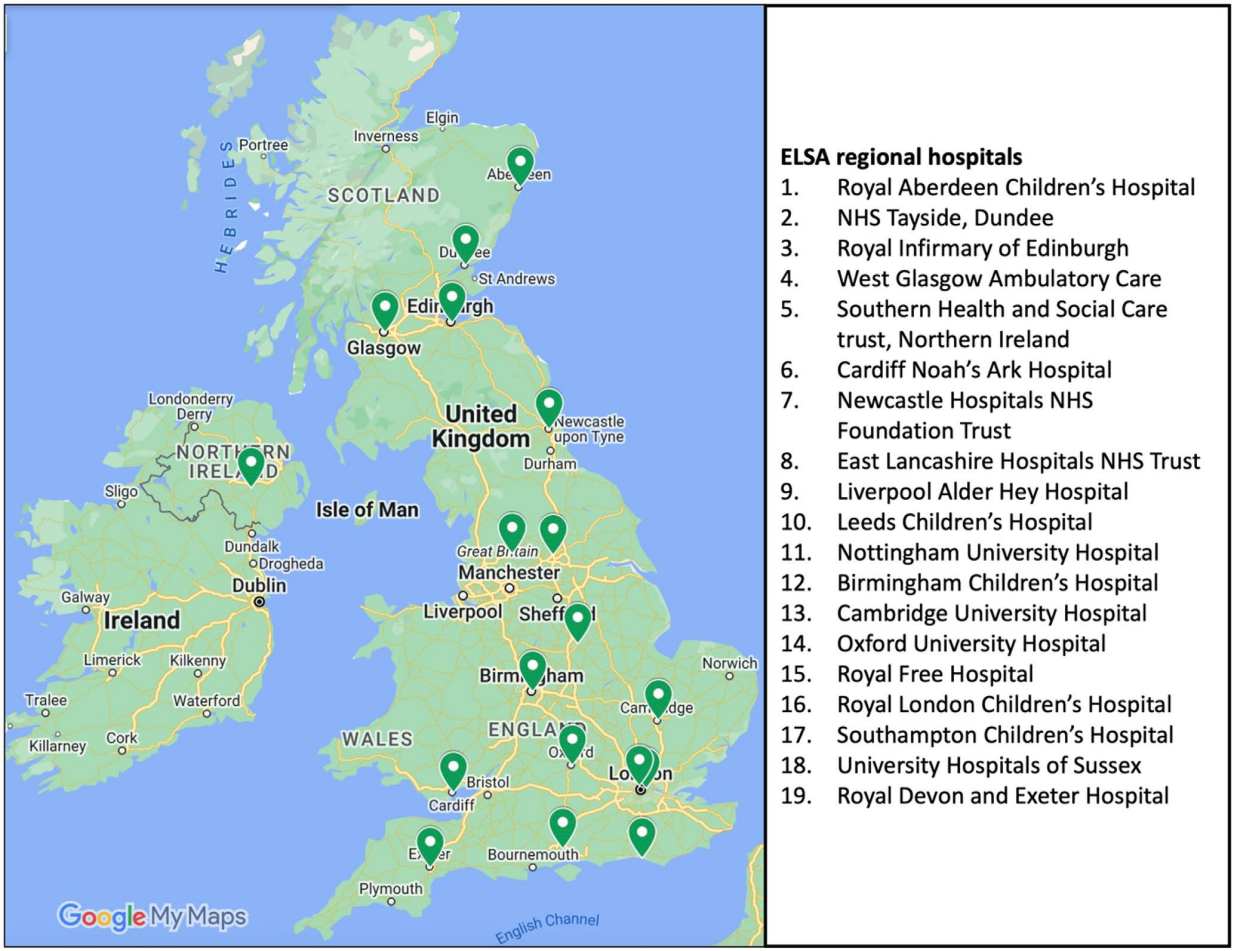
ELSA regional hospitals. ELSA hospitals offer venous autoantibody confirmation and OGTT. These are strategically located across all four UK nations to minimise travel burden for families. Image obtained from Google My Maps: https://www.google.com/maps/.

If the 3‐screen result is ≥20 IU, the parent is informed by telephone and invited to a regional paediatric hospital (Figure 2) for a venous sample to test for each of the four diabetes autoantibodies. Here, 4‐10 mL serum is obtained and an additional sample (up to 4 mL) for glycated haemoglobin (HbA1c) is processed locally. The autoantibody serum is stored at 4°C and returned within 24 h of sample collection to the CIS laboratory at the University of Birmingham.

Signs of haemolysis in the returned samples are recorded and serum is centrifuged and aliquoted on arrival and stored at 4°C prior to analysis. Serum is tested for anti‐GAD, anti‐IA‐2A, anti‐ZnT8 and anti‐insulin (IAA) autoantibodies using individual ELISA assays (Euroimmun IA2, GAD and ZnT8,^21^ and Orgentec Launch Diagnostics Insulin IgG ELISA).^22^

Parents of single autoantibody positive children are contacted by telephone, education offered, and results letter sent to the parent and GP. Children with two or more autoantibodies are informed by an ELSA clinician and referred for metabolic staging within 4 weeks.

#### 2.4.3 Metabolic testing

Metabolic testing is undertaken through an OGTT. A glucose solution, equivalent to 1.75 g of glucose per kg body weight (maximum 75 g), is ingested within 10 min. Venous glucose is collected at −20, 0, 30, 60, 90 and 120 min. Children are staged according to the American Diabetes Association (ADA) and International Society for Adolescent and Pediatric Diabetes (ISPAD) criteria (Table 1).^4^, ^5^ Stage 3 cases are referred to the local paediatric diabetes centre for management of clinical T1D. Stage 1 and 2 cases are invited for education and a results letter sent to the family and the GP. We recommend the international SNOMED code for ‘presymptomatic type 1 diabetes’ (5267060018) is included in the child's electronic health record.

**TABLE 1:** Staging criteria.

OGTT	Fasting glucose (mmol/L)	Intermittent glucose (mmol/L)	2‐h glucose (mmol/L)
Stage 1	<5.6	<11.1	<7.8
Stage 2	5.6–6.9	≥11.1 without symptoms	≥ 7.8–11.0
Stage 3	≥7.0	≥11.1 with symptoms	≥11.1

*Note*: Staging criteria for type 1 diabetes as recommended by the ADA and ISPAD. Stage 1 is defined as fasting glucose <5.6 mmol/L and 2‐h glucose <7.8 mmol/L. Stage 2 is defined as fasting glucose 5.6–6.9 mmol/L, 2‐h glucose 7.8–11.0 mmol/L and/or intermittent glucose ≥11.1 without symptoms. Stage 3 is defined as fasting glucose ≥7.0 mmol/L, 2‐h glucose ≥11.1 mmol/L and/or intermittent glucose ≥11.1 mmol/L with symptoms.

#### 2.4.4 Education

Parents with a child with one or more islet autoantibodies are invited to a one‐to‐one virtual (video or telephone) education session (30–60 min duration) delivered by a clinician. Discussion items include autoantibody and/or OGTT results, rate of progression and characteristic symptoms of stage 3 T1D. The session aims to counsel the family, address any concerns and alleviate anxiety. Parents receive an information sheet and are signposted to the INNOvative approach to understanding and arresting type 1 DIAbetes (INNODIA) study (https://www.innodia.eu/), or equivalent, for follow‐up and to the European and UK registries for children with presymptomatic T1D.

#### 2.4.5 Psychological assessment

For autoantibody positive children, parents complete an affect questionnaire after results notification (pre) and following education (post). The questionnaire consists of the shortened 6‐item State Anxiety Inventory (SAI) and the Hospital Anxiety and Depression Scale (HADS). A correction factor is applied to transform the short‐item SAI to a score representative of the full version (20 items).^23^ The SAI was validated in the US ASK study^24^ and the HADS is validated to detect anxiety and depression in adults and adolescents.^25^ In ELSA, children aged over 8 years also complete the HADS supervised by their parents. Psychological impact of screening (SAI and HADS) is compared before and after education.

If psychological distress is identified on the basis of these questionnaire scores, the family, parent and/or child are referred, with consent, to a clinical psychologist at Birmingham Women's and Children's Hospital (BWCH). The assessment and treatment at BWCH are undertaken virtually for families across the UK but would be referred to their local NHS service if ongoing care is required.

Single seropositive children are offered follow‐up autoantibody testing in the INNODIA study. Children with multiple autoantibodies are also followed up in the INNODIA study for HbA1c testing and OGTT. Children with clinical concerns, including osmotic symptoms or without access to home glucose monitoring may be referred to the local paediatric diabetes service. Referral to the BWCH presymptomatic T1D clinical service is available.

#### 2.4.6 Genetic testing

Parents can select genetic testing alongside the autoantibody screening. Genetic testing is performed on any remaining sample from the DBS card after elution for autoantibody detection. Excess DBS samples are sent to the University of Exeter and the genetic risk score (GRS2) is calculated.^26^ Genetic test results are intended for research purposes and families are not informed of the results.

### 2.5 Statistical analysis

#### 2.5.1 Feasibility assessment

The ELSA study aims to develop a system for identifying children with presymptomatic T1D who could benefit from the better glucose control associated with early detection, as well as future prevention trials or treatments. Testing such a system in a UK, NHS, public healthcare system is essential to understand facilitators and barriers to implementation. Table 2 outlines the planned assessments throughout the study, and the feasibility assessments include:

1. Number of participants recruited (% of target, demographics)
2. Recruitment modalities—relative uptake (% of total, demographics)
3. Study process failure rate (DBS/venous confirmation/OGTT)
4. Percentage uptake for venous confirmation (of those positive on DBS)
5. Percentage uptake for OGTT (of those ≥2Ab positive on venous confirmation)
6. Percentage uptake for education (of those autoantibody positive)
7. Percentage uptake for monitoring (of those autoantibody positive)
8. Withdrawals (recruitment approach, outcome/stage, demographics).

**TABLE 2:** ELSA Study visits and assessment summary.

ELSA visits	ELSA task / visit	(1) Recruitment and online consent	(2) DBS screening test	(3) Venous sample	(4) OGTT	(5) Affect questionnaire Pre‐education	(6) Education	(7) Affect questionnaire post‐education	(8) Feasibility questionnaire	(9) Qualitative interview
	Setting	Home, community or hospital	Home, community or hospital	Hospital	Hospital	Home	Home or hospital	Home	Home	Home or hospital
	Cohort	Parent and child	Child	Child	Child	Parent and child	Parent	Parent and child	Stakeholders	Parents Stakeholders
ELSA assessments	Measurements: Autoantibodies HbA1c OGTT		Autoantibody screening	Autoantibody confirmation and HbA1c	Staging					
	Questionnaires for Anxiety/depression					X		X		
	Feasibility								X	
	Acceptability									X

*Note*: ELSA tasks summarised in Figure 1 are performed at home, in community settings or at a regional hospital (Figure 2). ELSA measurements include autoantibody detection, glycated haemoglobin (HbA1c) and oral glucose tolerance testing (OGTT). The affect questionnaire assesses anxiety and depression in parents before and after the education session and children aged over 8 years can complete the questionnaire supervised by their parent. The feasibility questionnaire is administered to stakeholders with experience delivering the study. Qualitative interviews are conducted with parents and stakeholders to explore acceptability and implications of screening.

Feasibility of the different recruitment approaches (home testing, general practice, schools and hospitals) will be compared with respect to prespecified variables including participant demographics (parent and child's age, gender, ethnicity, deprivation level or family history of diabetes), and proportion DBS completion with sufficient sample. Proportional uptake for autoantibody confirmation, OGTT, education and follow‐up and reasons for withdrawal are recorded. A feasibility questionnaire is completed by staff at recruiting and follow‐up sites to understand the mechanics and practicalities of delivering ELSA screening clinics.

We will compare data set descriptive statistics from the study population, including demographics, recruitment approach and screening outcome. The quantitative data will be presented as mean +/− standard deviation (SD) and for qualitative data sets we will use Chi‐Squared Tests. We will use p‐value (corrected for T1D family history) as *p* < 0.05. Further descriptive analysis will include distributions of the measurements, missing value estimates and data visualisation. We will compare the efficacy (true positives, false positives and failure rate) of the RSR LTD 3‐screen with previous data^7^, ^20^ and if statistical analysis allows, we will estimate autoantibody seroprevalence for a UK cohort. Second, we will compare age, gender and other demographics between recruitment modalities and according to screening outcome. Finally, parents' and stakeholders' views from the qualitative interviews on the relative advantages and disadvantages of the recruitment modalities will be compared as part of the acceptability assessments.

Clinical outcomes include autoantibody seroprevalence in the UK population, prevalence of stage 1–3 and number of DKA cases (till ELSA end). Adverse and serious adverse events include loss of consciousness following blood taking and referral to psychology, respectively. Adverse events will be compared by recruitment modality and by outcome.

#### 2.5.2 Acceptability assessment

Parental and stakeholder acceptability is assessed following participation in the ELSA study. Acceptability is defined by Sekhon et al. as *‘a multi‐faceted construct that reflects the extent to which people delivering or receiving a healthcare intervention consider it to be appropriate, based on anticipated or experienced cognitive and emotional responses to the intervention’*.^27^ We aim to explore two dimensions of acceptability. The first relates to delivery of the programme, i.e., preferences for receiving information, performing the screening test, follow‐up testing, results giving and education, and the second encompasses the views and attitudes towards the intervention, i.e., whether screening aligns with the person's values and the benefits and harms of screening following notification of a screen positive result. The qualitative interviews aim to provide rich, insightful data on the acceptability of screening from a sample of at least 30 parents.

Acceptability will be assessed via semi‐structured interviews conducted in‐person, by telephone or video call (virtually). Interpreters are offered where English is not the first language.^28^ The topic guide for parents was developed from current acceptability literature, our peer‐reviewed publications, and consultation with patient and public involvement (PPI) members (Supplementary File 2). Interviews last 30–90 min and ask parents to share their experience of screening participation. Reasons for participation, reaction to the screening results, programme strengths and weaknesses, and emotional, social, cognitive and/or behavioural implications are explored. Attitudes towards general population screening, follow‐up, immunoprevention trials and treatment are sought. Interviews with stakeholders last 30–60 min and use a topic guide informed by the ‘Consolidated Framework for Implementation Research’ (CFIR) (Supplementary File 3).^29^

Interviews are audio recorded for transcription, independently coded by two qualitative researchers and thematically analysed.^30^ We will use the following frameworks for analysis: the Theoretical Domains Framework (TDF),^31^ our previously published Burdens of Screening’ (BoS) framework,^12^ and the Theoretical Framework for Acceptability (TFA).^27^ We will compare acceptability across ethnic groups, deprivation level, age of child, age of parent, recruitment setting and outcome (stage 1–2, single or multiple autoantibody positive, DBS negative). All demographic combinations may not be covered but transferable themes may nevertheless occur and we aim to sample until thematic saturation is reached. For stakeholders, the CFIR will be used as the analysis framework for systematic assessment of barriers and facilitators and to inform necessary adaptations for a future UK screening programme.

### 2.6 Patient and public involvement

Three co‐applicants and a PPI group, comprising parents and children, contributed to the grant application, design and implementation of ELSA. Parents and children co‐designed the information resources, website, videos and education session materials.

### 2.7 Ethics and dissemination

Measures have been put in place to address both the physical and psychological impacts of this research study. Whilst blood taking may cause some discomfort, only the minority (1–2%) require invasive testing and experienced phlebotomists are employed, anaesthetic creams applied and additional support available, e.g., play therapists. As with any screening test, there is a risk of false negative and false positive results,^20^ but clinical T1D symptom recognition is emphasised to all parents following ELSA participation. Finding out a child has presymptomatic T1D can be distressing; we aim to minimise this by providing results sensitively, counselling and offering psychological referral to mitigate mental health problems following results notification.

We plan to disseminate results at conferences and through peer‐reviewed publication.

### 2.8 Discussion and significance

The benefits of paediatric T1D screening include DKA prevention and giving families time to prepare.^6^ Screening also identifies individuals who can be offered novel therapies and trials for T1D prevention^4^; ELSA offers a UK model screening programme to serve these purposes. ELSA specifically seeks to identify optimal approaches to recruit and deliver general population screening for T1D in UK children, to facilitate access and meet the needs of diverse populations. Further, understanding the acceptability and implications of general population autoantibody screening, from both parents' and stakeholders' perspectives, is an international research priority and the ELSA study will formally explore this through qualitative interviews and measures of affect. The feasibility and acceptability assessments generated from ELSA will feed into future UK cost‐effectiveness analyses. This will provide the essential evidence to judge the benefits and harms for T1D screening and help inform future national screening recommendations.

## AUTHOR CONTRIBUTIONS

L.M.Q., R.P.D., S.M.G., A.R., J.G., D.S., A.A., G.G., R.O., S.F., O.B., I.L., F.B., C.M., A.L., C.G., F.Z., C.B., C.C., A.L., C.G., C.D., T.B. and P.N. have made substantial contributions to conception and design, or acquisition of data, or analysis and interpretation of data. L.M.Q., R.P.D., S.M.G., A.R., J.G., D.S., A.A., G.G., R.O., S.F., O.B., I.L., F.B., C.C., A.L., C.G., F.Z., C.B., C.D., T.B. and P.N. have been involved in drafting the manuscript or revising it critically for important intellectual content and gave final approval of the version to be published. L.M.Q., R.P.D., S.M.G., A.R., J.G., D.S., A.A., G.G., R.O., S.F., O.B., I.L., F.B., C.C., A.L., C.G., F.Z., C.B., C.D., T.B. and P.N. have agreed to be accountable for all aspects of the work in ensuring that questions related to the accuracy or integrity of any part of the work are appropriately investigated and resolved.

## FUNDING INFORMATION

The ELSA Study is co‐funded by Diabetes UK and Breakthrough T1D (formerly the Juvenile Diabetes Research Foundation) (grant code: 20_0006315). The ELSA Study also received funding from the European action for the Diagnosis of Early Non‐clinical Type 1 diabetes For disease Interception (EDENT1FI). This project is supported by the Innovative Health Initiative Joint Undertaking (IHI JU) under grant agreement No 101132379. The JU receives support from the European Union's Horizon Europe research and innovation programme, from The Leona M. and Harry B. Helmsley Charitable Trust, from Breakthrough T1D, from EFPIA, from COCIR, from Vaccines Europe, from EuropaBio and from MedTech. Additional funding is provided to associated UK partners through the UKRI (UK Research and Innovation) Guarantee Fund. The funders had no role in the study design; no role in data collection, analysis and interpretation of data; no role in the writing of the protocol paper; and no role in the decision to submit the paper for publication.

## CONFLICT OF INTEREST STATEMENT

LMQ, RPD, SMG, AR, JG, DS, AA, GG, RO, SF, OB, IL, FB, CC, AL, CG, FZ, CB, CD and PN have no conflict of interests to declare. TB is funded by the National Institute of Health Research (NIHR) as a senior investigator.

## Supporting information


**Supplementary File 1**—Parent’s informed consent form for the ELSA study.

**Supplementary File 2**—Interview topic guide for parents.

**Supplementary File 3**—Interview topic guide for professional stakeholders.
