# Supplementary material for: Protocol for a feasibility and acceptability study for UK general population paediatric type 1 diabetes screening—the EarLy Surveillance for Autoimmune diabetes (ELSA) study
Source: Diabet Med. 2024 Dec 2;42(5):e15490. doi: 10.1111/dme.15490 (PMC12006551; doi:10.1111/dme.15490)
Supplement: Supplementary file 1 — Parent’s informed consent form for the ELSA study. [file DME-42-e15490-s002.docx]

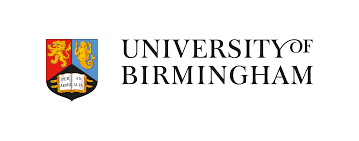

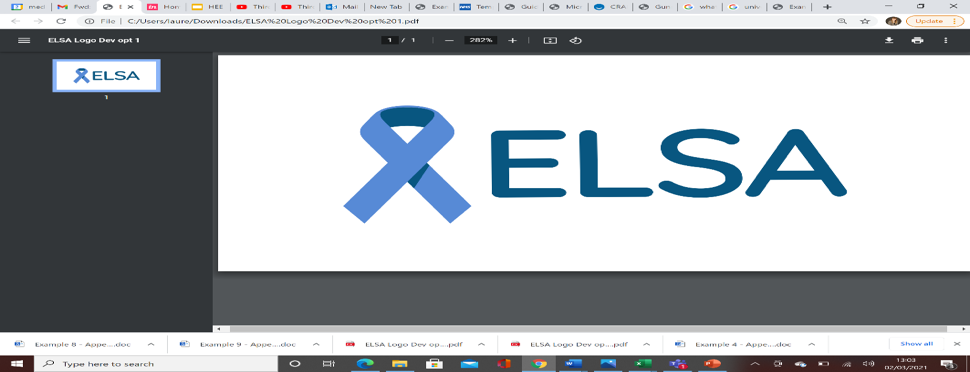


IRAS ID: 309252

Centre number: _____________________

Participant Identification Number for this trial: _____________________

**Parent/Guardian’s consent form**

**Title of Project: The ELSA Study**

**Please initial box**

1. I confirm that I have read the parent/guardian’s information sheet or completed the online information tool dated 26.07.2023 (version 3.0) for the above study. I have had the opportunity to consider the information, ask questions and have had these answered satisfactorily.
2. I understand that my child’s participation is voluntary and that I am free to withdraw my child at any time without giving any reason, without my child’s medical care or legal rights being affected. However, I also understand that once analysis has begun, it may not be possible to withdraw mine and my child’s anonymised data from the study.
3. I understand that relevant sections of my child’s medical notes and data collected during the study, may be looked at by individuals from the University of Birmingham, from regulatory authorities or from the NHS Trust, where it is relevant to my child taking part in this research. I give permission for these individuals to have access to my child’s records.
4. I understand that my personal details including my name, contact details, and demographic details, and my child’s personal details, demographic details, medical history and family history will be stored by the researchers for this study.
5. I understand that mine and my child’s data will be stored for 10 years after the study completes. A record of mine and my child’s personal details will be kept on a secure University of Birmingham server. Mine and my child’s paper records will be kept within a secure location in a locked office at the University of Birmingham. Results will be stored in a pseudo-anonymised form. Only lead and co-investigators will have access to this link.
6. I agree to the collection, transfer and storage of my child’s samples and/or relevant clinical data (as detailed in the information sheet), for use in this study and for up to 10 years, using a pseudo-anonymised study number. I understand that the samples will be analysed at the University of Birmingham or the Birmingham Children’s Hospital.
7. I give permission to be contacted by the study team to be informed of my child’s screening test results, by text message, phone call, email and/or letter. I understand that third parties, including Firetext, will be used to send me text messages to inform me of home-testing kit dispatch and my child’s antibody results, and DOCmail will be used to send me and my child’s GP a letter with my child’s screening test results. I agree to my personal data, including mobile phone number and address where relevant to the study, will be shared with these third parties.
8. I give permission for my child’s antibody tests and glucose results to be stored on NHS clinical systems.
9. I give permission for my child to be referred to the local paediatric diabetes service, if my child is found to have a new diagnosis of type 1 diabetes or if there are any clinical concerns.
10. I agree to take part in the ELSA type 1 diabetes screening programme.

**Optional: Yes No**

**Please initial box**

1. I agree that my child’s anonymised data and samples may be shared and transferred

to other research groups with whom we work in the UK, Europe, and the rest of the

world, (including the USA), including industrial partners, for collaborative, ethically approved research studies.

1. I agree to be contacted during the course of the study about collecting additional

information to understand acceptability of the ELSA screening programme.

1. If my child is confirmed positive for one or more antibodies from the venous sample,

I agree for my child’s details to be shared with INNODIA, and I understand INNODIA

will feed these results back to the ELSA study team, as detailed in the Participant Information Sheet.

1. I give permission for my child’s anonymous genetic material (DNA) to be tested for diabetes

risk genes as described in the parent’s information sheet.

1. I understand that the information held and maintained by the researchers at the

University of Birmingham and the NHS organisations involved in this study, may be

used to contact me about my child taking part in future ethically approved research or provide information about other studies relevant to my child’s screening test results. I give permission for my contact details being used for this purpose.

1. I agree to researchers from the University of Birmingham having access to my child’s

medical records in order to perform a follow-up study of my child’s data at a later date,

up to 10 years after the ELSA study.

1. I agree to provide my child’s NHS number (Community Health Index (CHI) number

in Scotland), for the study team to obtain long-term follow-up data

relevant to the study from my child’s medical records.

18. I agree to be contacted by email with study updates and information related to diabetes

screening, prevention and clinical trials.

19. Are you filling in this form with the help of an interpreter/translator? Y/N If you use

someone else's contact details please be aware that they will receive information about

your child and their screening test. This may include emails with questionnaires about your child's

medical history or results letters/calls.

Name of Parent/Guardian Date Signature

Name of Child you are consenting

Name of person taking consent Date Signature

**Thank you for completing the ELSA study screening programme consent form.**
