# Supplementary material for: Protocol for a feasibility and acceptability study for UK general population paediatric type 1 diabetes screening—the EarLy Surveillance for Autoimmune diabetes (ELSA) study
Source: Diabet Med. 2024 Dec 2;42(5):e15490. doi: 10.1111/dme.15490 (PMC12006551; doi:10.1111/dme.15490)
Supplement: Supplementary file 2 — Interview topic guide for parents. [file DME-42-e15490-s001.docx]

**Supplementary File 2:**

**Testing the feasibility and acceptability of EarLy Surveillance for Autoimmune diabetes:**

**The ELSA Study**

**Topic guide for screened participants**

**Interview Schedule for Families**

The following key questions will be used to trigger conversations with participants. Extra probing with follow-up questions will be determined by the initial responses given by participants and therefore the structure of each interview undertaken may vary.

**Introductions**

Thank you for agreeing to speak to me today.

Introduction of researcher

Family introductions

Reminder of study (interviewee will have received participant information sheet and signed consent form).

Confirmation that participant is happy to proceed.

Explanation of what happens to the data:

- Face to face and telephone interviews will be audio recorded. The audio recordings will be stored securely. For analysis the recording will be transcribed with anonymization. The recordings will then be deleted.

Reminder that the interviewee can stop the interview at any time and decide whether to restart or stop altogether

**Sections:**

**Section A) Screening story**

**Section B) Experiences of the screening programme**

**Section C) Impact of screening**

**Section D) Extra questions**

**Key:**

**Bold – sections**

*Italics – extra questions*

**This topic guide is for participants who took part in the ELSA 2 study**

**Screening story**

1. Could you start by telling us your story of going through this process?
2. What was your reaction to the screening test results at the time you received them?
3. What are your thoughts now about the screening test results?

**Experiences of the screening programme:**

1. How did you find out about the screening programme?
2. How did you decide to take part in the screening programme?
   1. Prompt: what was the most important factor in your decision to take part in the screening programme?
3. Could you talk us through your experiences of taking part in the screening programme?
   1. Were there any positive aspects?
   2. What about negative aspects? How could these be improved?
4. How did your child find the screening programme?
   1. Good parts
   2. Bad parts, areas for improvement?
   3. What factors might have influenced how they found it? E.g. child’s age, personality, comorbidities
5. Where did you have the DBS screening test done?
6. How did you find the DBS screening test for you/your child?
   1. Prompt: positive/negative aspects
7. If applicable: How did you/your child find the venous blood taking?
8. If applicable: How did you/your child find the OGTT?
9. If applicable: How did you/your child find the education session?
10. If applicable: What type of education session did you attend?
    1. Good parts
    2. Bad parts, areas for improvement?
11. If applicable: What were your thoughts on the educational materials?
    1. Good parts
    2. Bad parts, areas for improvement?
12. If applicable: How do you feel about your child entering a monitoring programme?
13. If applicable: How do you feel about your child entering trials that are testing new treatments to delay the start of, or prevent type 1 diabetes?

A medication has recently been licenced in the United States that is given to people at-risk of type 1 diabetes. The treatment, called Teplizumab, delays the start of type 1 diabetes and delays the need for insulin by 2-3 years on average. The treatment is an infusion given once a day for 14 days as a one-off course. The treatment has been licenced for children aged 8 years and above. This treatment is not currently licenced in the UK but is under review.

1. What is your initial reaction to this? Prompts to include:
   1. What information would you want to know?
   2. Would you want your child to receive this treatment or not?
   3. What would help you to decide?
   4. Mechanics of Teplizumab treatment:
      1. Side effects
      2. Infusion once a day over 14 days
      3. Any other concerns
   5. Considerations:
      1. Teplizumab licenced in the US, not yet available in the UK
      2. Teplizumab licenced for children aged 8 years and above
      3. Off-licence treatment for children aged younger than 8 years
      4. Teplizumab offers delay rather than prevention or cure
      5. Not all children at-risk would be eligible. Children not eligible could enter monitoring follow-up to determine if and when they become eligible.
      6. Barriers to accessing treatment
      7. Taking part in other research studies
   6. How do you think your child would react to this?
   7. Teplizumab is licenced for individuals aged 8-45 years. As a parent/guardian
      1. Would you want to be screened?
      2. Would you want to receive this treatment?

**Impact of screening**

1. What has been the impact of the screening programme on you/your child/your family?
   1. Positive
   2. Negative
   3. Optional: How do you see the future for you/your child/your family?
2. Have you told your child/friends/family about the screening test result?
   1. Do you think it's important to inform others about your child's result?
      1. If so/not, why? Who would you tell? If you child’s test was positive, would you think it is important to tell others
      2. If your child’s test was negative, would you think it is important to tell others?
3. Only if the family are known to have another child, ask:
   1. Do you think you would enter your other children into the screening programme?
4. Given what you know now, do you think you would make the same decision to enter your child into the screening programme?

**Closing**

1. Is there anything we haven’t talked about that you think is relevant?
2. Would you be interested in a follow-up interview?

**Extra questions**

1. To recruit families into this study, we used an ‘opt in’ approach. This means families have to actively consent to take part. Other approaches to taking consent include ‘opt out’ methods, which means that all children are automatically enrolled into the programme, unless the parent/guardian or child actively chooses to decline. What are your thoughts on an opt out screening programme for children?

Prompts: Positive, negative

- 1. What factors influence your views?
  2. How would the following affect your decision: accuracy of the test/ treatment availability/invasiveness of follow up testing/ number of children who will be identified as ‘at risk’ through participation in the programme

**Thank you for your time.**

**[Would you be interested in a follow-up interview?]**
