# Supplementary material for: Protocol for a feasibility and acceptability study for UK general population paediatric type 1 diabetes screening—the EarLy Surveillance for Autoimmune diabetes (ELSA) study
Source: Diabet Med. 2024 Dec 2;42(5):e15490. doi: 10.1111/dme.15490 (PMC12006551; doi:10.1111/dme.15490)
Supplement: Supplementary file 3 — Interview topic guide for professional stakeholders. [file DME-42-e15490-s003.docx]

**Supplementary File 3:**

**Testing the feasibility and acceptability of EarLy Surveillance for Autoimmune diabetes:**

**The ELSA Study**

**Topic guide for stakeholders**

**Interview Schedule for Stakeholders**

The following key questions will be used to trigger conversations with stakeholders. Extra probing with follow-up questions will be determined by the initial responses given by participants and therefore the structure of each interview undertaken may vary.

Start recording

**Introductions**

Thank you for agreeing to speak to me today.

Introduction of researcher

Reminder of study (interviewee will have received participant information sheet and signed consent form)

Confirmation that participant is happy to proceed.

Explanation of what happens to the data:

- Face to face and telephone interviews will be audio recorded. The audio recordings will be stored securely. For analysis the recording will be transcribed with anonymization. The recordings will then be deleted.

**Sections: CFIR domains and sub-domains:**

1. **Individual characteristics**
2. **Innovation domain**
   1. **Recruitment**
   2. **Practicalities and Mechanics of the study**
3. **Inner setting**
4. **Individual’s domain**
5. **Outer setting**
6. **Innovation and implementation outcomes**
7. **Views towards screening**

**Key:**

*Italics – extra questions*

**Individual characteristics – knowledge and experience with type 1 diabetes**

1. Stakeholder introduction
   1. Job role
   2. Specific role in the ELSA study

Which organisation do you work for?

1. What are your views on the way type 1 diabetes affects a child’s daily life?
   1. Prompts: Positive, negative?
   2. Age of child
   3. Personal experience
   4. Professional experience

**Innovation Domain – Thinking about your experiences of recruiting into the ELSA study in your organisation**

3. What was your experience of recruiting families/children from your organisation.

a. Prompt - How did you find the recruitment method(s)

b. Positive, negative

4. What were the reasons why a parent/guardian wanted to have their child screened?

5. And are you aware of any reasons why a parent/guardian chose not to have their child screened?

a. Prompts: anxiety, time, cost, burden, currently no treatment or cure

b. Reasons for drop-outs – travel burden, cost, anxiety

6. How did you find the online consent process.

a. Prompt – positive/negative experiences?

7. What facilitators/supporting factors helped recruitment in your organisation?

1. Prompts – funding, staff, team, family factors e.g. family history

8.Did you experience any barriers to recruitment in your organisation?

1. Prompts – funding, staff, team
2. What attempts were made to resolve these barriers? How successful were these attempts/was the barrier resolved?

9. Do you feel your organisations’ recruitment approaches were suitable to undertake recruitment and/or sampling for a diabetes screening programme?

10. Could you provide feedback on the resources/information about the study – information sheet, website, animations.

a. Prompts-was there enough, easily understandable information

b. Format of information: Written/Website/Face-to-face

II. Information for children – information sheet, videos

c. What factors supported informed consent?

d. Were there any difficulties or barriers to informed consent?

e. Any missing information for stakeholders / parents / children?

f. Any areas for improvement?

11. Did you feel adequately knowledgeable to be able to answer parents and children’s questions in relation to the study

1. Prompt - Do you feel you had adequate training to deliver the study in your setting

I**nnovation domain - Practicalities and Mechanics of the study - Thinking about your experiences of implementing/delivering the ELSA study in your organisation.**

1. How did you as a stakeholder/your organisation (school/general practice) find the ELSA screening programme overall?
2. Design
3. Adaptability
4. Complexity
5. Trialability
6. How does ELSA compare to current practice? Other alternatives?
7. Acceptability
8. How did you find the finger prick test?
   1. How did you/stakeholders find the screening test?
   2. How did the parents find the test
   3. Where did you perform the screening test – hospital, school, community, other
   4. What supporting factors were associated with DBS testing?
   5. What difficulties were associated with DBS testing?
9. What do think could be the most appropriate setting for the screening test?
   1. Prompts:
   2. GP surgery/Childhood vaccination programme e.g. MMR, HPV/School/Local community centre/Home testing/Any other?
   3. Are there any advantages/disadvantages of any of these locations - Which is best?
   4. *Extra question: How do you feel about a child being tested without the parent/guardian being present (schools) or without a health care professional being present (home)?*
10. How did you find the further testing - venous confirmation and OGTT
    1. Stakeholders’ perspective
    2. How did the parents find the venous confirmation and OGTT
    3. How did the children/young people find the venous confirmation and OGTT
    4. What supporting factors were associated with further testing?
    5. What difficulties were associated with further testing?
11. Can you describe your experiences of communicating with families/parents/children in relation to results?
    1. Prompts: -
       1. Remotely or in person
       2. Letter/e-mail/text message/phone call
       3. By a member of the research team/ your GP/ other healthcare professional
    2. Within what time frame would you expect to receive the results: days/weeks/months
    3. Your experience of results giving
    4. Your experience of education
12. How did you find the education and support for ELSA participants identified with Pre-T1D or at-risk?
    1. Virtual or in-person, individual or group session with families in a similar situation to yours
13. How soon after finding out the test result – days, weeks?
14. Suggested areas for improvement?
15. What support for ELSA participants identified with pre-T1D/at-risk should be provided?
16. How did you find the clinical follow-up (INNODIA) for ELSA participants identified with pre-T1D or at-risk?
    1. Should this be research or clinical care?
    2. What were the supporting factors to families entering follow-up?
    3. What were the difficulties for families entering follow-up?
    4. Suggested areas for improvement?
17. Thinking about different parts of the ELSA study, what do you think is most and least appealing about the ELSA screening programme?

Prompts: Screening test, further testing, education, monitoring

1. Can you think of any supportive factors for families’ participation in the ELSA screening programme?
   1. Personal factors – family history diabetes, age of child, personality of child, child’s comorbidities, personality of parents
   2. Structural factors – geography,
   3. Situational factors
2. Can you think of any factors which prohibited families’ participation in the ELSA screening programme?
   1. Personal factors
   2. Structural factors
   3. Situational factors
   4. What about local communities within which the organisation sits and what these communities’ needs are and their reactions to screening
3. For families with screen-positive results, were you aware of any implications/burden for families’ participating in the ELSA screening programme?
   1. Emotional
   2. Cognitive – understanding the risk status
   3. Financial
   4. Social – professional or family life
   5. *Can you think of any implications of a child knowing they have Pre-T1D or are at risk?*

**Inner setting – Thinking about your organisation**

1. **Structural characteristics** – Thinking about implementation of the ELSA screening programme overall, please describe how the infrastructure in your organisation/research site interacted with, supported, or impeded implementation.
   1. Prompts – interacted, supported, impeded?
   2. Physical infrastructure
   3. Information Technology infrastructure
   4. Work infrastructure - Organisation of tasks and responsibilities
   5. Relational connections - General staffing levels
2. **Compatibility -** How did the ELSA screening programme integrate into your organisations’ existing work processes?
   1. What (if any) ELSA screening programme components or processes needed to be changed to fit into existing hospital/research site work processes?
   2. What (if any) hospital/research site work processes needed to be changed to fit the ELSA screening programme?
3. **Relative priority -** What was the priority of implementing and/or delivering ELSA compared to other initiatives in your organisation?
4. **Incentive systems –** What kinds of incentives and/or disincentives may influence implementation and/or delivery of the ELSA study
   1. E.g. Recruitment targets?
5. **Mission alignment** - How does implementing and/or delivering [innovation] align with your organisations’ mission and goals?
6. **Available resources -** To what extent were necessary resources available to implement/deliver the ELSA screening programme?
   1. Funding
   2. Space
   3. Materials and equipment
7. **Access to knowledge and information -** What kinds of training and guidance were available to support implementation/delivery of the ELSA screening programme?
   1. What information or training was missing?

**Individuals domain – Thinking about the team you worked with on implementation of the ELSA study in your organisation**

1. **Relational connections -** What is your working relationship like with colleagues you work closely with in your organisation/research site that were also involved in ELSA implementation?
   1. With colleagues you interact with in other areas of the hospital/research site?
   2. To what extent do people work in teams?
2. **Communications -** Hospitals/research sites typically communicate using multiple methods, e.g., email, staff meetings, word-of-mouth: How did you typically hear about ELSA study matters?
   1. What information would you like to have that is not usually shared?
3. **Roles -** In your organisation, who fulfilled the following position and what was their role?
4. Leaders – high/mid/opinion
5. Implementation facilitators / leads / team members / other support
6. Research participants
7. **Personal experience -** How did you, personally find implementation of the ELSA study
   1. Supporting factors to your work on the ELSA study
   2. Difficulties or challenges you personally faced?

**Outer setting – thinking about the wider implementation of the ELSA study**

1. **Critical incidents -** Were there any (recent) unanticipated events that influenced implementing the ELSA screening programme?
   1. If yes: How did this event influence implementation or delivery of the ELSA screening programme?
2. **Local attitudes and conditions -** What level of support (if any) was needed from the central (University of Birmingham) ELSA study team to implement or deliver the ELSA screening programme?
   1. Level of external support required
   2. What kinds of attitudes do people within Birmingham ELSA team have toward you/your organisation?
   3. How did these attitudes encourage or discourage Birmingham ELSA team from supporting implementation and/or delivery of the ELSA study?
   4. What were the working conditions and how did these conditions enable or hinder the ELSA Birmingham team from supporting implementation and/or delivery of the ELSA screening programme in your organisation/research site?
   5. Any areas for improvement?
3. How did you find the REDCap research database for the Elsa study?
   - 1. Supporting factors with the REDCap research database?
     2. Difficulties or challenges with the REDCap database?
4. **Partnerships and connections -** To what extent did your organisation have established connections to entities outside your organisation, relevant to the ELSA screening programme?
   1. Please describe these connections.
   2. To what extent do you network or exchange information with colleagues outside of your hospital/research site? Please describe these relationships.
5. **Policies and laws -** What kinds of legislation, regulations, professional group guidelines/recommendations, or accreditation standards helped or hindered implementation or delivery of the ELSA screening programme?
6. **Financing -** What types of funding were needed from outside your hospital/research site to implement and/or deliver the ELSA screening programme?
   1. To what extent was this funding available?
7. **Pressures (societal/market) -** What kinds of pressures outside your hospital/research site influenced implementation and/or delivery of the ELSA screening programme?
   1. Prompts - social/societal/public domain pressures influenced implementation and/or delivery of the ELSA screening programme?
8. **Performance pressure -** What kinds of performance measures influenced implementation and/or delivery of the ELSA screening programme?
   1. E.g. Clinical Research Network – Recruitment targets
   2. Local audits

**Innovation and implementation outcomes – thinking about successful implementation**

1. **Innovation outcomes (outer setting, effectiveness and reach) –** Overall, how effective/successful do you think the ELSA screening programme was?
   1. Personal perspective
   2. From the perspective of the implementation team in your organisation
   3. From research participants’ perspective
   4. From Key decision makers’ perspective
2. **Innovation outcomes (inner setting, effectiveness and reach) –** How effective/successful was implementation of the ELSA screening programme in your organisation?
   1. Personal perspective
   2. From the perspective of the implementation team in your organisation
   3. From research participants’ perspective
   4. From Key decision makers’ perspective
3. **Implementation outcomes -** What overall impact did the ELSA screening programme have?
   1. Positive
   2. Negative
   3. Success stories
   4. Difficult case examples
4. Would you recommend the study to other organisations like yours?
5. Is there anything that could be improved/changed to make this study better?

**Views toward screening:**

1. What are your views about screening children for type 1 diabetes in the UK?
2. Prompts: positives, negatives
3. General population or targeted (familial) screening
4. Any prior experience of screening?
5. Facilitators to rolling out a national screening programme?
6. Barriers to rolling out a national screening programme?
7. Have your views changes following participation in the ELSA study?
8. What are you views towards a licensed treatment/ immunoprevention trials:
9. Teplizumab
10. Immunoprevention trials
11. Benefits
12. Harms
13. *If a child was having a blood test to find out their risk of type 1 diabetes, what are your thoughts on screening for other conditions they may have or be at risk of, like coeliac disease? If we could do these tests on the same blood sample provided.*

*Prompts: Positive, negative*

o *What factors would your decision depend on?*

**Is there anything else that you would like to add?**

**Would you be interested in a follow-up interview?**

**Thank you for your time.**
